# MEK1/2 Signalling establishes an alloreactive effector T cell program through SMAD4 during cardiac allograft rejection

**DOI:** 10.1002/ctm2.70865

**Published:** 2026-10-27

**Authors:** Jiulu Zhao, Shuan Ran, Sijie Zhou, Yuan Li, Wang Zhan, Longyong Lai, Jikai Cui, Jiahong Xia, Jie Wu

**Affiliations:** ^1^ Department of Cardiovascular Surgery, Union Hospital, Tongji Medical College Huazhong University of Science and Technology Wuhan China; ^2^ Center for Translational Medicine, Union Hospital, Tongji Medical College Huazhong University of Science and Technology Wuhan China; ^3^ Institute of Translational Medicine, Tongji Medical College Huazhong University of Science and Technology Wuhan China; ^4^ Hubei Key Laboratory of Biological Targeted Therapy, Union Hospital, Tongji Medical College Huazhong University of Science and Technology Wuhan China; ^5^ Key Laboratory of Organ Transplantation, Ministry of Education; NHC Key Laboratory of Organ Transplantation Key Laboratory of Organ Transplantation Chinese Academy of Medical Sciences Wuhan China

**Keywords:** heart transplant rejection, MEK/ERK signalling, SMAD4, T cells

## Abstract

**Background:**

Acute allograft rejection is largely dependent on T cell‐driven immune responses; however, the molecular programs that establish and maintain effector T cell states during rejection remain incompletely defined.

**Methods:**

Single‐cell transcriptomic analysis was conducted to characterize the activation of signalling pathways in T cells during acute allograft rejection. The function of MEK1/2 signalling was evaluated both in vitro and in vivo using mice with targeted deletion of *Mek1/2* in T cells and pharmacological inhibition. Phosphoproteomic analysis, immunofluorescence staining, and western blotting were conducted to investigate downstream signalling mechanisms, particularly those involving SMAD4.

**Results:**

Effector CD4 and CD8 T cells exhibited enhanced MEK/ERK pathway activation throughout the rejection process. MEK1/2 inhibition, either via genetic deletion or pharmacological blockade, attenuated T cell activation and effector activity, leading to reduced rejection and prolonged graft survival. Phosphoproteomic analysis identified SMAD4 as a key downstream effector of MEK1/2 signalling. Mechanistically, MEK1/2 deficiency reduced SMAD4 nuclear translocation in activated T cells, accompanied by diminished effector T cell responses.

**Conclusions:**

MEK1/2 signalling regulates T cell activation and effector phenotypes via SMAD4 nuclear translocation in heart transplant rejection. Therapeutic intervention targeting this pathway may contribute to alloimmune response regulation and improve transplant outcomes.

## Graphical abstract

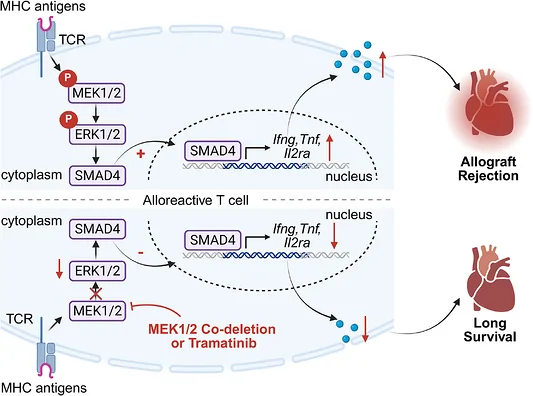

Highlights

MEK1/2 signalling drives an alloreactive effector T‐cell program that promotes cardiac allograft rejection.

MEK1/2 signalling promotes SMAD4 nuclear translocation to sustain T‐cell effector function.

Pharmacological targeting of MEK1/2 restrains alloimmune activation and prolongs graft survival, supporting the MEK1/2–SMAD4 axis as a potential therapeutic target.

## 1 INTRODUCTION

T cells are central to adaptive immunity and crucial for orchestrating alloimmune responses following organ transplantation.^1^, ^2^ Antigen exposure triggers naïve T cell activation and clonal expansion, resulting in the generation of effector subsets responsible for orchestrating immune responses.^3^, ^4^, ^5^ Activated T cells infiltrating the allograft secrete proinflammatory cytokines, directly contributing to tissue damage and acute rejection.^6^ Despite significant progress in immunosuppressive therapy, T cell–mediated alloimmune responses remain a key challenge to long‐term allograft survival, highlighting the need to further elucidate the mechanisms governing T cell activation and effector function.^2^, ^7^, ^8^ Engagement of the TCR initiates T cell activation, triggering a cascade of intracellular signalling pathways that coordinate transcriptional and metabolic reprogramming.^9^, ^10^ Despite the characterization of early signalling events downstream of TCR engagement, the mechanisms sustaining T cell activation and maintaining effector function remain poorly defined. In particular, how upstream signalling pathways integrate with transcriptional programs to support effector T cell responses is yet to be elucidated.

The MEK/ERK pathway is a fundamental downstream component of TCR signalling that manages multiple programs of T cell biology, including activation, proliferation, differentiation, and survival.^11^, ^12^ After TCR engagement, MEK1/2 is rapidly activated and serves as a critical kinase transmitting intracellular signals to ERK1/2, thereby linking extracellular stimulation to transcriptional responses.^13^, ^14^ Furthermore, MEK/ERK signalling has been implicated in metabolic reprogramming, particularly in promoting glycolysis to support the energetic and biosynthetic demands of activated T cells.^15^ Beyond its role in early activation, MEK/ERK signalling may also participate in the maintenance of T cell functional states and effector programs, suggesting a broader role in sustaining immune responses.^16^ However, the contribution of this pathway to T‐cell‐mediated alloimmune responses, particularly in the context of cardiac transplantation, has not been sufficiently characterized.

Emerging evidence indicates that signalling pathways such as MEK/ERK may interact with transcriptional regulators to influence T cell function. As a central mediator of TGF‐β signalling, SMAD4 forms complexes with phosphorylated SMAD2/3 and translocates to the nucleus for transcriptional regulation.^17^, ^18^, ^19^ In T cells, SMAD4 has been implicated in differentiation, cytokine production, and lineage stability.^20^, ^21^ Notably, SMAD4‐dependent transcriptional programs have been associated with effector and regulatory T cell (Treg) responses, demonstrating its context‐dependent roles in immune regulation.^22^, ^23^ Beyond its canonical function, SMAD4 activity can be modulated by upstream signalling pathways. ERK signalling reportedly facilitates SMAD4 nuclear translocation and enhances its transcriptional activity, indicating a potential association between MEK1/2 signalling and SMAD4‐mediated gene regulation.^24^ This interaction provides a plausible mechanism by which extracellular signals are integrated with transcriptional programs for T cell response regulation. However, whether this regulatory axis contributes to T cell function during alloimmune responses remains unclear.

Herein, MEK1/2 signalling in T cell‐mediated acute cardiac allograft rejection is characterized, and the molecular mechanisms involved are revealed. A T cell‐specific *Mek1/2* double‐deficient mouse model was used to examine the cell‐autonomous functions of MEK1/2 in T cells. Subsequently, functional assays and phosphoproteomic analysis were conducted to investigate the effects of MEK1/2 deficiency on T cell activation, effector responses, and signalling cascades, specifically focusing on the MEK1/2–SMAD4 axis. Collectively, these results shed light on the signalling networks that orchestrate T cell function in transplantation and provide potential avenues for more targeted immunomodulatory strategies.

## 2 MATERIALS AND METHODS

Detailed descriptions of the experimental materials and procedures are available in the Supporting Information Materials and Methods.

### 2.1 Mice

Male C57BL/6 (B6; 219) and BALB/c (211) mice, aged 6–12 weeks, were obtained from Charles River (Beijing, China). *Cd4‐Cre^+^* (B6. Cg‐Tg[Cd4‐cre]1Cwi/BfluJ, #022071) mice were obtained from The Jackson Laboratory (USA). *Map2k1* and *Map2k2* floxed mice on a C57BL/6 background were generated using CRISPR/Cas9 technology by Shanghai Model Organisms. These mice were subsequently bred with *Cd4‐Cre^+^* mice to obtain *Map2k1^fl/f^Map2k2^fl/fll^Cd4‐Cre^+^* mice.^25^ Genotyping primers are listed in Table S2. All procedures involving animals were conducted at the Laboratory Animal Center of Huazhong University of Science and Technology and received prior approval from the Institutional Animal Care and Use Committee of Huazhong University of Science and Technology (IACUC No. 5041). Mice were housed under specific pathogen‐free (SPF) conditions. Anaesthesia was induced and maintained with 2% isoflurane throughout the surgical procedures. Euthanasia was performed by CO_2_ inhalation.

### 2.2 Murine heterotopic cervical heart transplantation

The heterotopic heart transplantation model in mice was constructed based on a previous study.^26^ The donor BALB/c mice were anesthetized with 2% isoflurane and positioned supine. We made a midline incision to expose the thoracic cavity and harvested the heart following the ligation of the major vessels. The donor heart was immediately immersed in 4°C heparinized saline for preservation. Recipient mice on a C57BL/6 background were anesthetized, and heterotopic cervical heart transplantation was performed. The donor aorta and pulmonary artery were anastomosed to the recipient common carotid artery and external jugular vein, respectively. Graft function was assessed daily by palpation. Successful engraftment was defined as a palpable heartbeat within 72 h after surgery (success rate > 90%). Graft rejection was defined as the complete loss of palpable graft contractions and subsequently verified by direct inspection during dissection.

For MEK1/2 inhibition, male BALB/c mice (age: 6–8 weeks) were used as donors, and male wild‐type C57BL/6 mice (age: 8–12 weeks) served as recipients. From day 0 to 15 after transplantation, trametinib (HY‐10999, MedChemExpress) was administered by oral gavage once daily. Trametinib was formulated in a vehicle containing 10% dimethyl sulfoxide (DMSO) and 90% corn oil (v/v) and administered orally at 3 mg/kg. Control mice were administered an equal volume of vehicle. Subsequent analyses were performed at the indicated time points after the transplantation.

### 2.3 In vitro T cell isolation and activation

Naïve T cells were purified from the spleens of WT or *Mek1/2*‐tKO mice using the Pan T cell Isolation Kit II (130‐095‐130, Miltenyi Biotec). For activation, purified naïve T cells were plated in 96‐well round‐bottom plates and stimulated with plate‐bound anti‐CD3 (5 µg/mL; 553057, BD Biosciences) and anti‐CD28 (1 µg/mL; 553294, BD Biosciences) following 3 days of exposure to recombinant IL‐2 (10 ng/mL; 554424, BD Biosciences).

### 2.4 Flow cytometry and cell sorting

Single‐cell suspensions were first incubated with anti‐CD16/32 antibody (clone 93; BioLegend) at 4°C for 20 min to block nonspecific Fc receptor binding. Surface staining was performed in a FACS buffer containing fluorochrome‐conjugated antibodies for 30 min at 4°C. Dead cells were identified and excluded using the Zombie Aqua Fixable Viability Kit (423102, BioLegend). Following surface staining, intracellular Ki‐67 and FOXP3 were detected using the intracellular/transcription factor staining kit (00‐5523‐00; eBioscience). Intracellular cytokine production was evaluated following restimulation with PMA (M4647, Abmole) and ionomycin (407951, Sigma‐Aldrich) in the presence of GolgiStop Protein Transport Inhibitor containing monensin (554724; BD Biosciences). Cells were subsequently fixed, permeabilized, and stained for the indicated intracellular cytokines. To assess phosphorylated MEK1/2 and ERK1/2, cells were stimulated with PMA (1 µg/mL) and ionomycin (1 µg/mL) at 37°C for 15 min, followed by fixation with Phosflow Lyse/Fix Buffer (554049, BD Biosciences) at room temperature for 15 min. The cells were permeabilized using Phosflow Perm Buffer III (558050, BD Biosciences) and stained with the corresponding antibodies at room temperature for 1 h. Annexin V and 7‐AAD staining (640934; BioLegend) were carried out according to the manufacturer's instructions. Table S1 presents a list of all antibodies used in this study. Flow cytometry data were collected using either a BD LSRFortessa X‐20 or Sony 7000 flow cytometer and analyzed with FlowJo software (version 10.8.1; BD Biosciences).

### 2.5 ELISpot assay

The mouse IFN‐γ precoated ELISpot kit (2210001; Dakewe) was used in accordance with the manufacturer's instructions. On day 6 following cardiac transplantation, spleens were harvested from the indicated groups and dissociated into single‐cell suspensions. Cell viability was evaluated by trypan blue exclusion, and the cell suspension was subsequently adjusted to 2 × 10^6^ cells/mL. Following mitomycin C treatment (HY‐13316, MCE), stimulator cells were mixed with responder splenocytes at a 1:1 ratio and seeded into precoated ELISpot plates. Cells were incubated at 37°C for 16 hours. Following incubation, plates were washed and sequentially incubated with biotinylated anti‐IFN‐γ detection antibody and streptavidin–HRP. Following additional washing steps, TMB substrate solution was added, and the spots were developed in the dark for 15–30 min. The reaction was terminated by rinsing with deionized water, followed by air‐drying. Spots were enumerated using a Mabtech IRIS FluoroSpot/ELISpot reader, and the results were reported as spot‐forming units (SFU) per 10^6^ splenocytes.

### 2.6 Statistical analysis

All statistical analyses were performed using GraphPad Prism (version 10.1.2). Unless otherwise indicated, data were presented as mean ± standard deviation (SD). For two‐group comparisons, a two‐tailed unpaired Student's *t*‐test was employed for normally distributed data with equal variances and Welch's *t*‐test for those with unequal variances. For non‐normally distributed data, the Mann–Whitney *U* test was used. For multigroup comparisons, one‐ or two‐way analysis of variance (ANOVA) was used for normally distributed data and the Kruskal–Wallis test for nonnormally distributed data, followed by Dunnett's or Sidak's post hoc multiple comparisons tests, as appropriate. Statistical significance is indicated as follows: ns, not significant; *p < .05; **p < .01; *** *p* < .001; ****p < .0001.

## 3 RESULTS

### 3.1 Single‐cell transcriptional profiling identifies MEK–ERK‐associated transcriptional programs in effector T cells during cardiac transplant rejection

To dissect the heterogeneity and functional characteristics of alloreactive T cell populations in heart transplant rejection, previously published single‐cell transcriptomic datasets generated from a murine heart transplantation model were comprehensively analyzed (Figure S1A,B).^27^ After 6 days of transplantation, live CD45^+^ immune cells were obtained from the spleens of syngeneic recipients (Iso_spleen) and from the spleen (Allo_spleen) and cardiac allografts (Allo_graft) of allogeneic recipients for library preparation and sequencing. In the syngeneic transplantation group, complete MHC compatibility between donors and recipients prevented the occurrence of acute rejection. Consequently, only a small number of CD45^+^ immune cells were recovered from the grafts (Iso_graft), which were insufficient for single‐cell library construction and therefore excluded from subsequent analyses. After sample integration and quality control, T cells were extracted and analyzed using unsupervised clustering and dimensionality reduction, resulting in the identification of six T cell clusters based on established marker genes (Figure 1A).^25^ Among these clusters, Naïve_CD4 and Naïve_CD8 demonstrated high expression of genes characteristic of resting T cells (*Sell, Ccr7*); Teff_CD4 and Teff_CD8 showed high expression of effector‐associated genes (*Ifng*, *Pdcd1*, *Gzmb*); NK‐T cells were characterized by high expression of *Ncr1*; and Treg cells preferentially expressed *Foxp3* (Figure 1B). Comparative profiling of T cell subset distributions across the three groups revealed that the Iso‐spleen was predominantly composed of naïve T cell populations, whereas effector immune subsets, including Teff_CD4, Teff_CD8, and NK‐T cells, were markedly enriched within the Allo_graft group (Figure 1C,D; Figure S1C).

**FIGURE 1:**
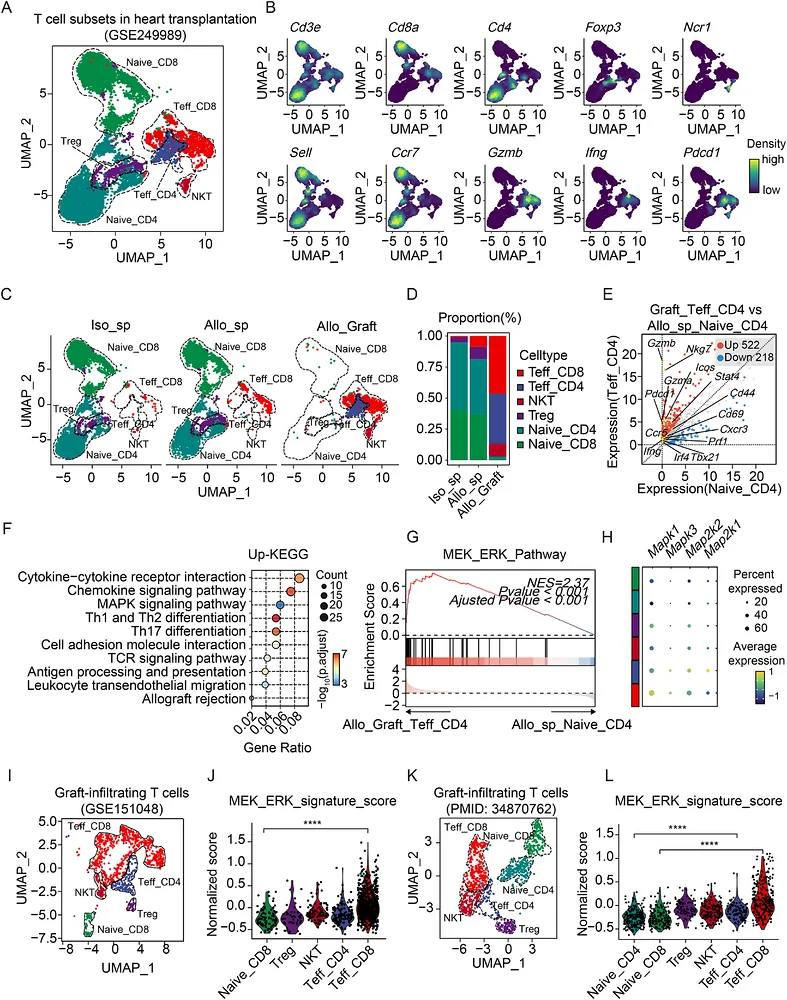
scRNA‐seq identifies enhanced MEK/ERK signalling in effector T cells during acute heart allograft rejection. (A) UMAP visualization of T cell clusters (GSE249989). (B) UMAP density plots illustrating marker gene expression across the T cell clusters. (C) UMAP visualization showing the distribution of T cell clusters across the Iso_spleen, Allo_spleen, and Allo_graft groups. (D) Bar plots showing the proportions of the six T cell clusters among the different groups. (E) Scatter plot illustrating differentially expressed genes (DEGs) between Allo_graft Teff_CD4 and Allo_spleen Naïve_CD4 T cell subsets. Upregulated (red dots) and downregulated (blue dots) DEGs are highlighted. (F) Dot plot showing significantly enriched Kyoto Encyclopedia of Genes and Genomes pathways among DEGs between Allo_graft Teff_CD4 and Allo_spleen Naïve_CD4 T cell subset clusters. (G) Gene set enrichment analysis plots of MEK/ERK pathway‐related genes in Teff_CD4 cells. (H) Bubble plot showing the expression of four key genes of the MEK_ERK pathway in six T cell clusters. (I) UMAP visualization of five T cell clusters infiltrating cardiac grafts from a published dataset (GSE151048). (J) Violin plots showing the MEK_ERK pathway scores across five T cell clusters. (K) UMAP visualization of six T cell clusters infiltrating cardiac grafts from the published dataset (PMID: 34870762). (L) Violin plot showing the MEK_ERK pathway scores of six T cell clusters.

To obtain further insight into molecular programs promoting effector T cell activation during acute rejection, effector and naïve T cells from the Allo_graft and Allo_spleen groups were systematically compared. Comparative transcriptomic analysis revealed substantial transcriptional alterations in Teff_CD4 cells relative to Naive_CD4 cells, including 522 upregulated and 218 downregulated differentially expressed genes (Figure 1E). KEGG pathway analysis of upregulated genes revealed marked enrichment in classical functional pathways, such as cytokine–cytokine receptor interaction, chemokine signalling, as well as Th1 and Th17 differentiation. Notably, the MAPK signalling pathway, which has previously received less attention, was significantly enriched (Figure 1F). Consistent results were observed in Teff_CD8 T cells, where upregulated genes were enriched in both classical functional pathways and the MAPK signalling pathway (Figure S1D,E). To further assess MEK–ERK‐related transcriptional signatures across T cell subsets, gene set enrichment analysis was performed. Compared with Naïve_CD4 and Naïve_CD8 T cells from the Allo_spleen group, Teff_CD4 and Teff_CD8 populations in the Allo_graft group showed significant enrichment of MEK–ERK‐associated gene sets (Figure 1G; Figure S1F). Moreover, DotPlot visualization of key MEK–ERK pathway genes (*Mapk1*, *Mapk3*, *Map2k1*, and *Map2k2*) showed that their expression was predominantly enriched in Teff CD4 and Teff CD8 T cells (Figure 1H). These transcriptional analyses suggest an association between effector T cell states and MEK–ERK‐related transcriptional programs during allograft rejection.

To validate these findings in independent datasets, two additional published scRNA‐seq datasets were analyzed.^28^ After consistent quality control and clustering, five and six T cell subsets were identified in graft‐infiltrating populations, which predominantly consisted of Teff_CD4 and Teff_CD8 T cells, respectively, corroborating the aforementioned findings. Notably, MEK–ERK pathway scores were significantly higher in graft‐infiltrating Teff_CD4 and Teff_CD8 T cell subsets (Figure 1I–L). Collectively, these independent datasets further support the enrichment of MEK–ERK‐associated transcriptional programs in effector CD4^+^ and CD8^+^ T cells in rejecting cardiac allografts, implicating MEK–ERK signalling in effector T cell responses during allograft rejection.

### 3.2 The MEK/ERK pathway is markedly activated in effector T cells during heart transplant rejection

To validate the findings from the previous bioinformatics analyses, a fully MHC‐mismatched murine model of acute heart transplant rejection was developed (Figure 2A). On day 6 after transplantation, spleen and cardiac allografts were harvested for immune cell isolation and subsequent flow cytometry analysis to evaluate MEK/ERK signalling activity. The phosphorylated forms of MEK1/2 (p‐MEK1/2) and ERK1/2 (p‐ERK1/2) were markedly increased in the allogeneic group, with the highest levels observed in graft‐infiltrating CD44^+^CD4^+^or CD44^+^CD8^+^T cells (Figure 2B–E). Immunofluorescence staining further confirmed the presence of p‐MEK1/2 and p‐ERK1/2 within graft‐infiltrating T cells (Figure 2F). These results indicate that effector T cells undergo substantial activation of the MEK–ERK pathway during acute rejection of cardiac allografts.

**FIGURE 2:**
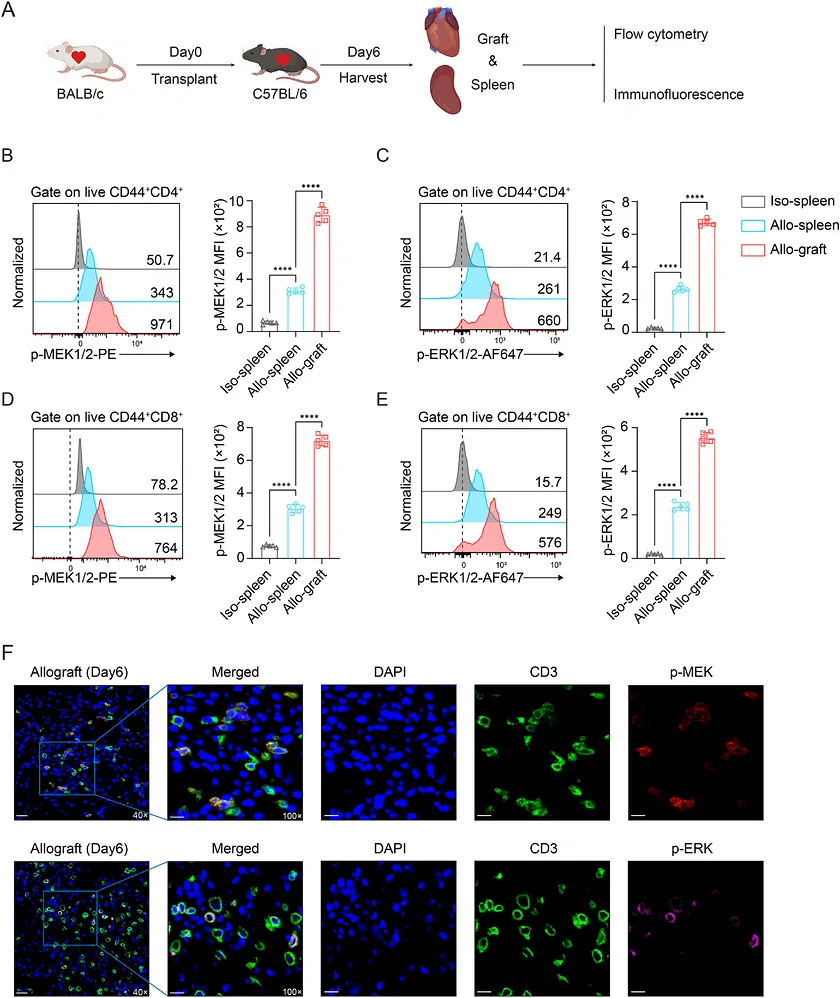
The MEK/ERK pathway is highly activated in effector T cells during acute heart allograft rejection. (A) Experimental design for the analysis of grafts and spleens on day 6 following cardiac transplantation using flow cytometry and immunofluorescence. (B) Representative flow cytometry plots and quantification of p‐MEK1/2 expression in Teff CD4^+^ cells isolated from spleens and allografts. (C) Representative flow cytometry plots and quantification of p‐ERK1/2 expression in Teff CD4^+^ cells isolated from spleens and allografts. (D) Representative flow cytometry plots and quantification of p‐MEK1/2 expression in Teff CD8^+^ cells isolated from spleens and allografts. (E) Representative flow cytometry plots and quantification of p‐ERK1/2 expression in Teff CD8^+^ cells isolated from spleens and allografts. (F) Representative immunofluorescence images showing p‐MEK, p‐ERK, and CD3 expression in allografts on day 6 post‐transplantation (CD3, green; p‐MEK/p‐ERK, red; DAPI, blue). Scale bars: 40× (20 µm); 100× (10 µm). All data are representative of at least three independent experiments. Data are expressed as mean ± SD (*n* = 5). Statistical significance for panels (B–E) was determined via one‐way ANOVA, followed by Sidak's multiple‐comparisons test. ns, not significant (*p* > .05); **p < .01; ***p < .001; ****p < .0001.

### 3.3 Deletion of T cell‐specific *Mek1/2* attenuates heart transplant rejection in mice

Next, we generated T cell‐specific *Mek1/2* double‐knockout mice (hereafter referred to as *Mek1/2*‐tKO mice) and used them in heart transplantation experiments (Figure 3A). Gene deletion was validated at the genomic DNA and protein levels, as previously reported.^29^ Subsequently, allograft survival outcomes were further analyzed following T cell‐specific deletion of *Mek1/2*. Notably, recipient *Mek1/2*‐tKO mice showed significantly prolonged allograft survival, with graft beating maintained for over 100 days (Figure 3B). Staining using haematoxylin and eosin showed substantial accumulation of inflammatory cells in grafts from littermate control mice on day 6 after transplantation. Contrarily, *Mek1/2*‐tKO recipients exhibited minimal inflammatory infiltration on day 6, and only mild, localized inflammation with limited myocardial structural disorganization was observed even on day 100 (Figure 3C). Consistently, the rejection scores were significantly reduced in *Mek1/2*‐tKO mice compared with those in littermate controls on day 6 after transplantation (Figure 3D).

**FIGURE 3:**
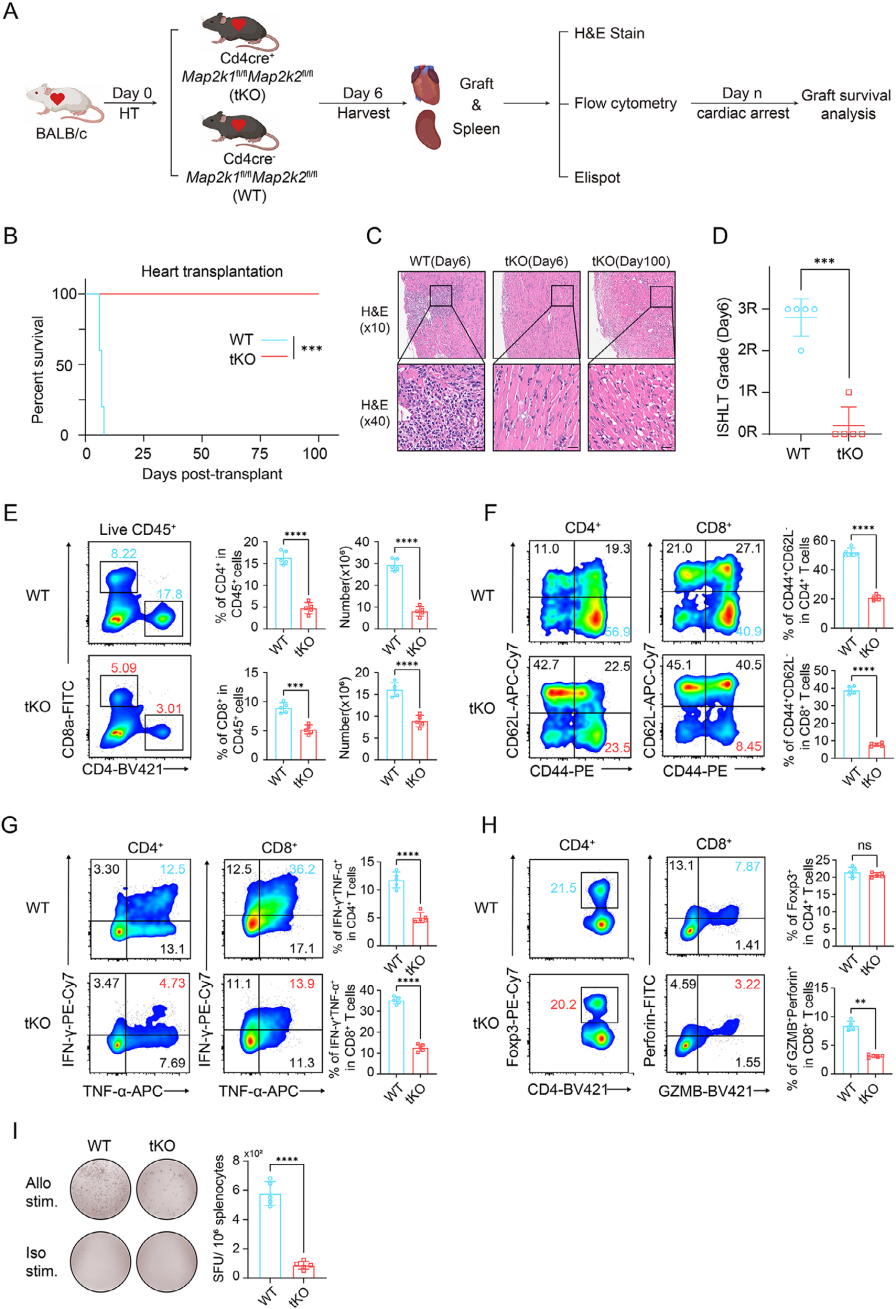
T cell–specific *Mek1/2* deficiency attenuates heart transplant rejection in mice. (A) Experimental flowchart. BALB/c hearts were transplanted into WT or *Mek1/2*‐tKO mice, after which spleens and cardiac allografts were collected for subsequent analyses. Graft survival was monitored daily by palpation until complete loss of cardiac contractility. (B) Survival curve of cardiac allografts in WT or *Mek1/2*‐tKO recipient mice. (C) H&E pathological section of cardiac allografts from WT or *Mek1/2*‐tKO recipient mice following transplantation; scale bar: 50 µm. (D) Cellular rejection scores of cardiac allografts in WT or *Mek1/2*‐tKO recipients, evaluated according to the 2004 ISHLT guidelines. (E) Representative pseudocolor images and quantification of the proportion and absolute number of CD4^+^ and CD8^+^ T cells in the spleens of WT or *Mek1/2*‐tKO recipient mice on day 6 after transplantation. (F) Flow cytometry plots and quantification of CD44^+^CD62L^−^ cells within CD4^+^ and CD8^+^ T cells from the indicated groups. (G) Flow cytometry pseudocolor images and quantification of TNF‐α^+^IFN‐γ^+^ in the WT and *Mek1/2*‐tKO groups. (H) Flow cytometry analyses and quantification of FOXP3^+^ cells within CD4^+^ T cells and GZMB^+^Perforin^+^ cells within CD8^+^ T cells from WT and *Mek1/2*‐tKO mice. (I) Representative IFN‐γ ELISpot images and quantification of spot‐forming units (SFU) per 10^6^ splenic T cells from WT or *Mek1/2*‐tKO recipient mice on day 6 after transplantation. All data are representative of at least three independent experiments. Data are expressed as mean ± SD (*n* = 5). Statistical significance was determined using the log‐rank test for (B), the Mann–Whitney U test for (D, H), and an unpaired two‐tailed Student's *t*‐test for (E–G, I). ns, not significant (*p* > .05); **p < .01; ***p < .001; ****p < .0001.

Flow cytometry was conducted to determine how *Mek1/2* deficiency influences T cell responses. T cells isolated from recipient mouse spleens were assessed for subset distribution, cytokine production, and cytotoxic molecule expression (gating strategy presented in Figure S2). T cell–specific *Mek1/2* deletion markedly reduced the proportion and absolute numbers of CD4^+^ and CD8^+^ T cells within recipient mouse spleens (Figure 3E), without significantly affecting Treg frequency (Figure 3H, left). On day 6 after transplantation, the frequency of activated CD44^+^CD62L^−^ T cells markedly decreased across the CD4^+^ and CD8^+^ T cells in *Mek1/2*‐tKO recipients (Figure 3F). Functionally, *Mek1/2* deficiency considerably reduced dual TNF‐α^+^IFN‐γ^+^ effector T cell populations among CD4^+^ and CD8^+^ T cells (Figure 3G), along with cytotoxic GZMB^+^perforin^+^ CD8^+^ T cell populations (Figure 3H, right). Consistent with these findings, ELISpot assays showed impaired alloreactive IFN‐γ secretion in T cells derived from *Mek1/2*‐tKO mice (Figure 3I). MEK1/2 deficiency in recipient mice significantly attenuated heart transplant rejection by restraining T cell expansion, activation, and effector cytokine production.

### 3.4 *Mek1/2* deficiency impairs T cell proliferation, activation, and effector function in vitro

To further assess the cell‐intrinsic contribution of MEK1/2 to T cell function, naïve CD4^+^ and CD8^+^ T cells isolated from WT and *Mek1/2*‐tKO spleens were subjected to in vitro anti‐CD3/CD28 stimulation (Figure 4A). After 3 days of in vitro culture, *Mek1/2*‐tKO mice showed a reduced proportion of CD44^+^CD25^+^ activated subsets within both CD4^+^ and CD8^+^ compartments (Figure 4B), along with reduced PD‐1 expression (Figure 4C). Furthermore, the effector function was compromised, with lower frequencies of TNF‐α^+^IFN‐γ^+^ coproducing cells and a significant reduction of GZMB^+^perforin^+^ CD8^+^ T cells (Figure 4D,E). Subsequently, T cell proliferative capacity and survival were assessed. *Mek1/2* deficiency markedly reduced the proportion of Ki67^+^ cells in CD4^+^ and CD8^+^ T cells (Figure 4F), whereas apoptotic rates remained comparable between groups, as indicated by similar Annexin V^+^7AAD^+^ populations between the groups (Figure 4G). Overall, these findings suggest that the loss of MEK1/2 signalling intrinsically restrains T cell expansion, activation, and effector function in vitro.

**FIGURE 4:**
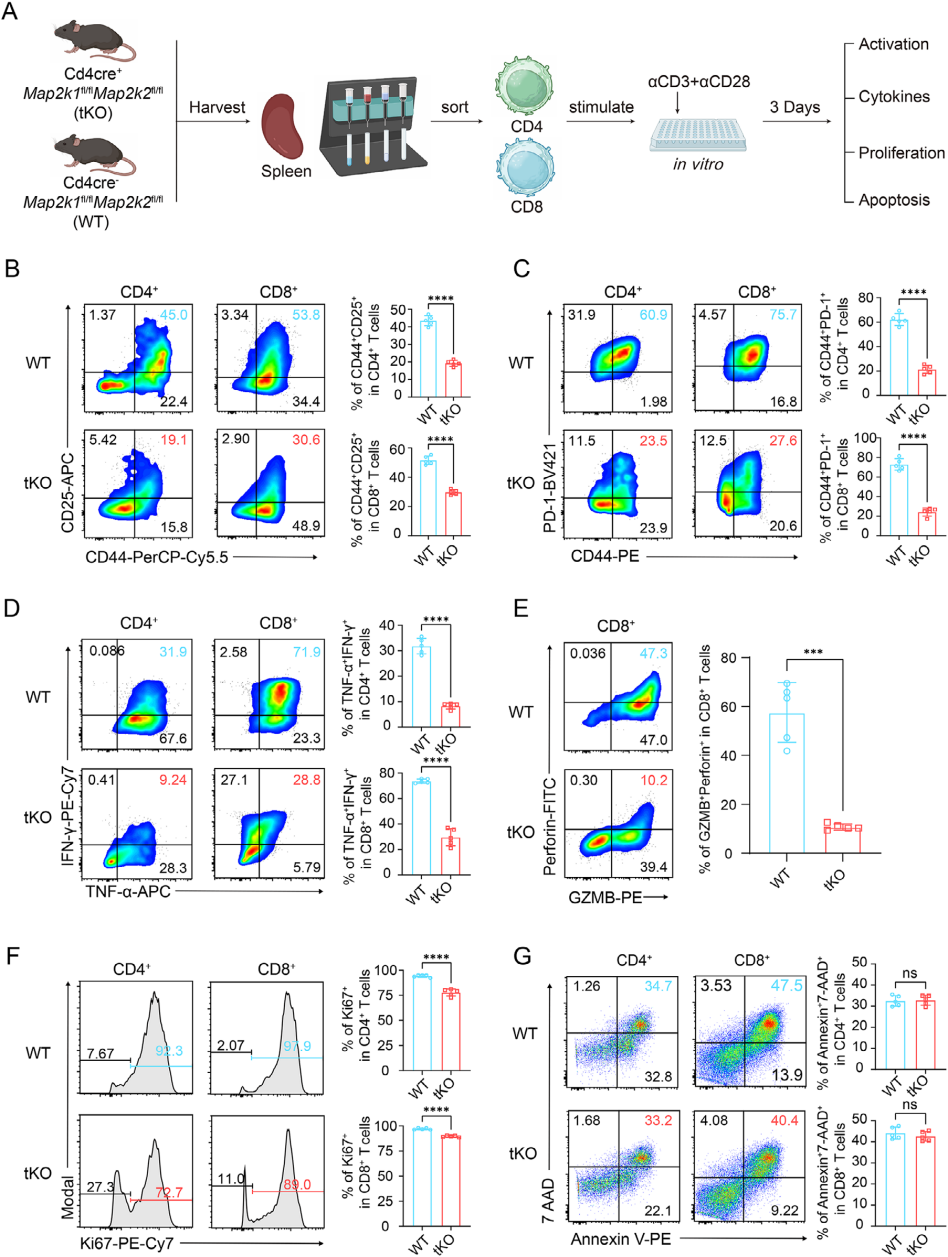
*Mek1/2* deficiency impairs T cell proliferation, activation, and effector function in vitro. (A) In vitro experimental design. Naïve T cells were isolated from the spleens of WT or *Mek1/2*‐tKO mice and stimulated with anti‐CD3/CD28 for 3 days, followed by downstream functional assays. (B, C) Representative flow cytometry plots and quantification of CD44 coexpression with CD25 (B) or PD‐1 (C) in T cells from the indicated groups. (D, E) Flow cytometry analyses and quantification of TNF‐α^+^IFN‐γ^+^ (D) or GZMB^+^Perforin^+^ (E) T cells in the WT and *Mek1/2*‐tKO groups. (F, G) Flow cytometry histograms and quantification of Ki‐67^+^ cells (F) and Annexin V^+^7‐AAD^+^ cells (G) in CD4^+^ and CD8^+^ T cells from WT and *Mek1/2*‐tKO mice. All data are representative of at least three independent experiments. Data are expressed as mean ± SD (*n* = 5). Statistical significance was determined using Welch's *t*‐test for (D, E) and an unpaired two‐tailed Student's *t*‐test for (B, C, F, G). ns, not significant (*p* > .05); **p < .01; ***p < .001; ****p < .0001.

### 3.5 MEK1/2 signalling regulates T cell activation and function via SMAD4 nuclear translocation

On this basis, this study aimed to investigate the molecular mechanisms by which MEK1/2 signalling controls T cell activation and function. Phosphoproteomic analysis of WT and *Mek1/2*‐tKO T cells was performed (Figure 5A). Principal component analysis showed a distinct separation between the two groups, with good intragroup consistency (Figure 5B). Using a cutoff of |log_2_(fold change)| > 1 and adjusted *p *< .05, 617 upregulated and 217 downregulated phosphorylation sites in *Mek1/2*‐tKO T cells were identified compared with those in WT controls (Figure 5C). The analysis focused on the potential downregulated signalling. Reactome pathway enrichment analysis revealed significant suppression of multiple pathways, with prominent enrichment of the downregulated SMAD2/3: SMAD4 active pathway (R‐MMU‐2173795), which drew our attention (Figure 5D). SMAD2, SMAD3, and SMAD4 are key mediators of TGF‐β superfamily signalling that regulate cell growth, fibrosis, and immune responses via phosphorylation cascades.^30^, ^31^ SMAD4 serves as a central hub that promotes nuclear translocation of activated SMAD complexes and cooperates with transcriptional cofactors to regulate target gene expression.^32^ Recent studies have demonstrated that ERK‐dependent phosphorylation facilitates SMAD4 nuclear translocation, promoting the transcription of cytotoxic genes, such as GZMB and perforin, in CD8^+^ T cells.^24^ Based on these findings, it was hypothesized that SMAD4 acts downstream of MEK1/2 in regulating T cell function.

**FIGURE 5:**
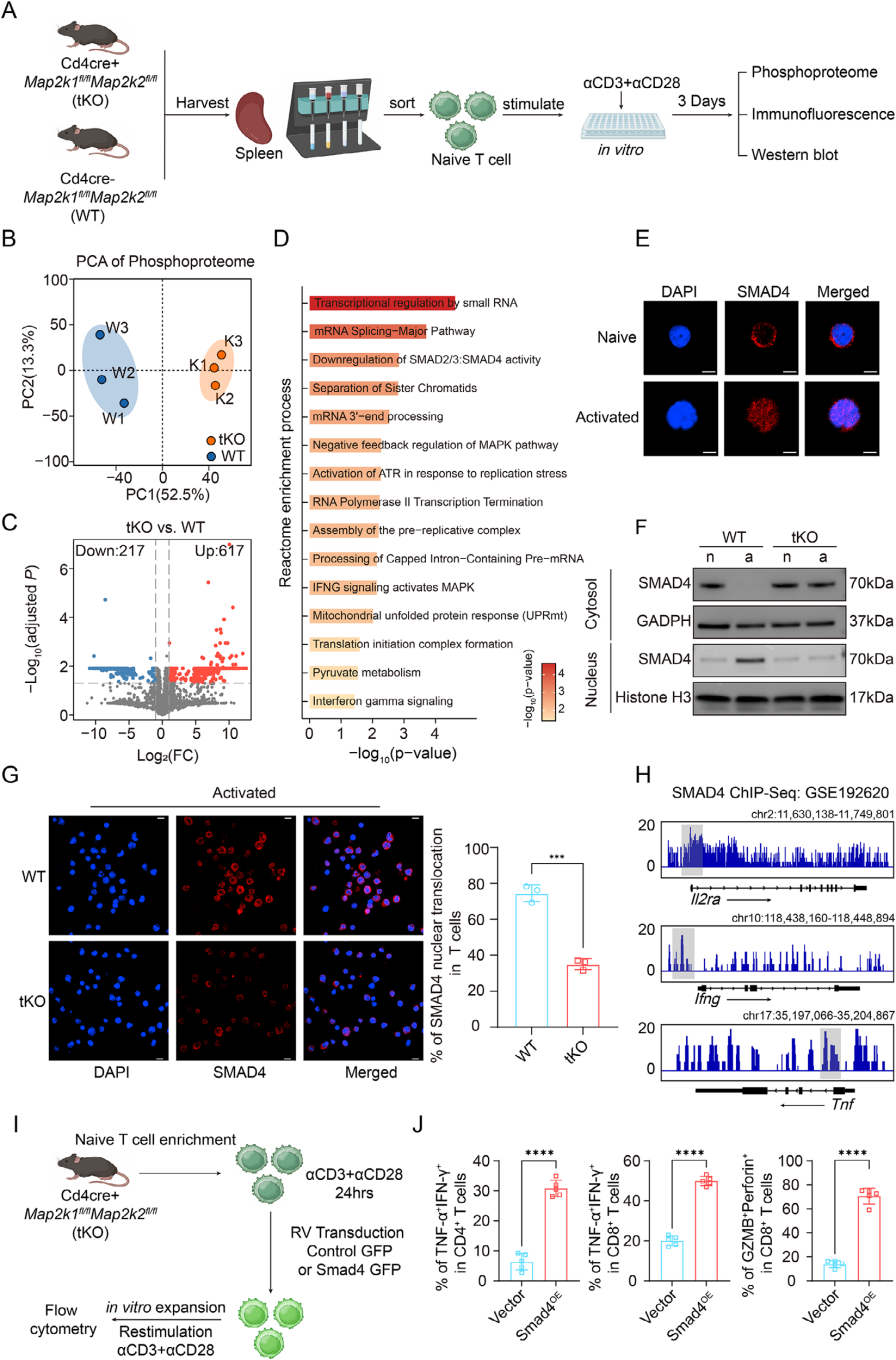
Mek1/2 regulates T cell activation and function via SMAD4 nuclear translocation. (A) Schematic of the experimental workflow. (B) Principal component analysis of phosphoproteomics in WT or *Mek1/2*‐tKO T cells following a 3‐day in vitro stimulation. (C) Volcano plot showing differentially phosphorylated protein sites between the WT and *Mek1/2*‐tKO T cells. Upregulated (red) and downregulated (blue) sites are highlighted. (D) Bar plot showing the Reactome pathway enrichment of downregulated phosphorylated sites. (E) Representative immunofluorescence images of SMAD4 localization in naïve and T cells stimulated in vitro for 3 days (SMAD4, red; DAPI, blue). Scale bar: 10 µm. (F) Representative Western blot images of SMAD4 in the cytoplasmic and nuclear fractions of naïve cells or T cells subjected to 3‐day in vitro stimulation. (G) Representative immunofluorescence images and bar graphs of the nuclear translocation levels of SMAD4 in WT or *Mek1/2*‐tKO cells after a 3‐day in vitro stimulation (SMAD4, red; DAPI, blue); scale bar: 10 µm; (*n* = 3). (H) Integrative Genomics Viewer (IGV) tracks showing normalized SMAD4 binding at the *Il2ra*, *Ifng*, and *Tnf* gene loci in T cells after a 48 h in vitro stimulation (SMAD4 ChIP‐Seq: GSE192620). (I) Experimental scheme and (J) flow cytometric quantification of TNF‐α^+^IFN‐γ^+^ or GZMB^+^Perforin^+^ T cells (*n* = 5) transduced with retroviruses encoding *Samd4* (Smad4^OE^) or control GFP (vector). All data are representative of at least three independent experiments. Data are expressed as mean ± SD. Statistical significance for panel (G, J) was determined using an unpaired two‐tailed Student's *t*‐test. ns, not significant (*p* > .05); **p < .01; ***p < .001; ****p < .0001.

To validate this hypothesis, we initially confirmed SMAD4 nuclear translocation in WT T cells following anti‐CD3/CD28 stimulation after 3 days via immunofluorescence. SMAD4 was cytoplasmic in naïve T cells but translocated to the nucleus upon activation (Figure 5E). Western blotting of cytoplasmic and nuclear fractions revealed efficient SMAD4 nuclear accumulation in WT cells, which was strongly impaired in *Mek1/2*‐tKO T cells (Figure 5F).

Immunofluorescence further demonstrated reduced nuclear localization in the knockout group (Figure 5G). Reanalysis of publicly available SMAD4 ChIP‐seq data (GSE192620) further revealed SMAD4 occupancy at several immune‐related genes, including *Il2ra, Ifng, and Tnf* (Figure 5H), supporting a potential role for SMAD4 in the transcriptional regulation of T cell effector responses.^24^ Next, we tested whether SMAD4 functionally mediates the impaired effector response associated with MEK1/2 deficiency. Therefore, we transduced *Mek1/2‐tKO* T cells with an empty control retrovirus or a retrovirus encoding *Smad4*. Retroviral SMAD4 overexpression (Smad4^OE^) increased the frequencies of TNF‐α^+^IFN‐γ^+^ CD4^+^ and CD8^+^ T cells, as well as GZMB^+^Perforin^+^ CD8^+^ T cells (Figure 5J). Collectively, MEK1/2‐dependent T cell effector function is associated with SMAD4 nuclear translocation, and restoring SMAD4 expression partially rescues the effector phenotype.

### 3.6 Pharmacological inhibition of MEK1/2 with trametinib suppresses acute heart transplant rejection

Furthermore, whether pharmacological MEK1/2 blockade protects against acute cardiac allograft rejection was investigated. The recipient mice were treated with corn oil or the MEK1/2 inhibitor trametinib daily from day 0 (Figure 6A). Trametinib markedly prolonged allograft survival (Figure 6B). Histopathological analysis on day 6 revealed extensive inflammatory infiltration and myocardial damage in the control group, whereas trametinib‐treated grafts exhibited only mild inflammation and preserved tissue architecture, alongside lower rejection scores (Figure 6C). Flow cytometry further demonstrated that trametinib markedly reduced cytokine production by recipient T cells, as shown by reduced proportions of TNF‐α^+^IFN‐γ^+^ cells in both CD4^+^ and CD8^+^ T cell subsets (Figure 6D). These results align with the genetic model, indicating that MEK1/2 inhibition suppresses effector T cell responses and effectively mitigates acute heart transplant rejection.

**FIGURE 6:**
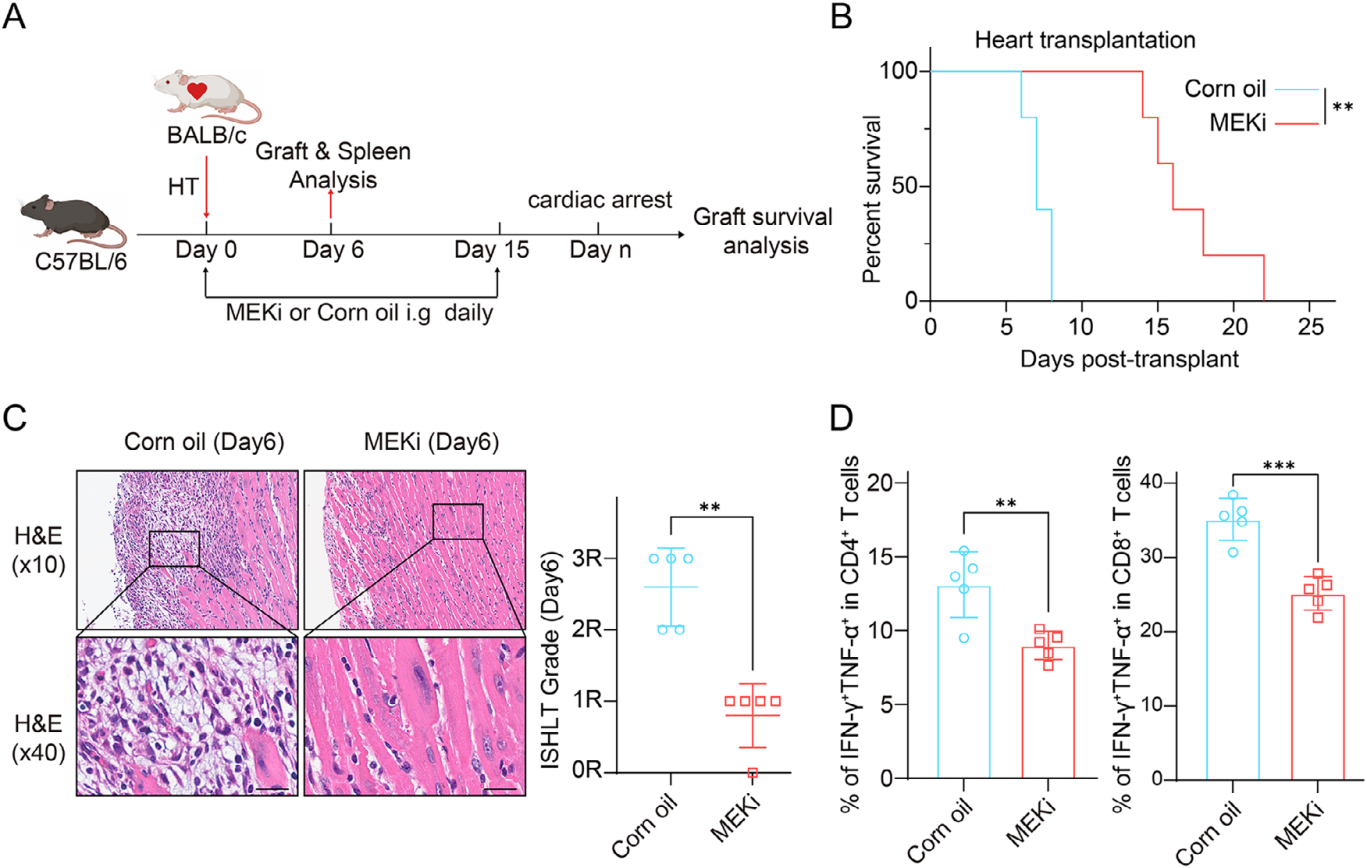
Pharmacological inhibition of MEK1/2 with trametinib suppresses acute heart transplant rejection. (A) Experimental design of MEK1/2 inhibitor treatment. WT C57BL/6 mice underwent BALB/c cardiac allografts on day 0 and were treated daily with MEKi (trametinib) or corn oil. Grafts and spleens were harvested on day 6 after transplantation. Graft survival was monitored daily by palpation until complete loss of cardiac contractility. (B) Survival curve of allografts in recipient mice. (C) Representative H&E pathological sections and scatter plots of allografts in recipients treated with trametinib or corn oil on day 6; scale bar: 25 µm. (D) Bar graphs showing the proportions of IFN‐γ^+^TNF‐α^+^ CD4^+^ and CD8^+^ T cells in the spleen of recipients treated with trametinib or corn oil. All data are representative of at least three independent experiments. (B–D) All data are representative of at least three independent experiments. Data are expressed as mean ± SD (*n* = 5). Statistical significance was determined using the log‐rank test for (B), the Mann–Whitney *U* test for (C), and an unpaired two‐tailed Student's *t*‐test for (D). ns, not significant (*p* > .05); **p < .01; ***p < .001; ****p < .0001.

### 3.7 Activation of the MEK‐ERK pathway is associated with allograft rejection in human heart and kidney transplantation

To translate the above findings into a human disease context, we examined MEK–ERK signalling in publicly available transcriptomic datasets from cardiac and renal allograft biopsies (Figure 7A). In both datasets, the scores of the MEK–ERK pathway were significantly higher in allograft rejection samples than in stable controls (Figure 7B). These findings indicate that increased MEK–ERK pathway activity is associated with the rejection state across different types of solid‐organ transplantation. Next, we examined the association between MEK–ERK pathway activity and graft outcomes using Kaplan–Meier survival analysis with log‐rank testing. Recipients with higher MEK–ERK pathway scores had inferior graft outcomes, whereas those with lower pathway activation had prolonged allograft survival (Figure 7C). Overall, these findings indicate that increased MEK–ERK pathway activity is related to allograft rejection and unfavourable graft outcomes, supporting MEK/ERK signalling as a likely treatment target in transplantation.

**FIGURE 7:**
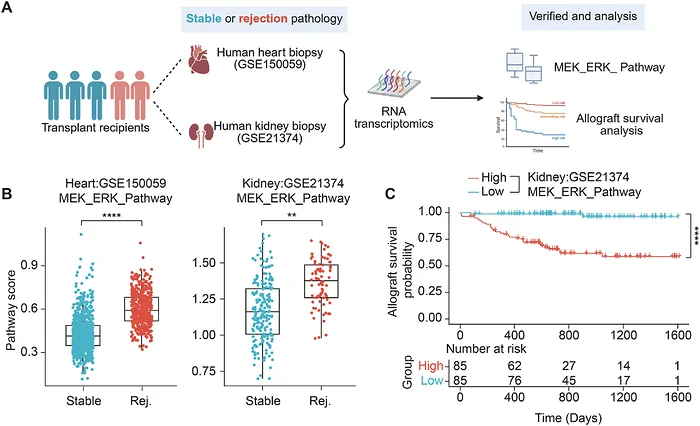
Activation of the MEK–ERK pathway is associated with allograft rejection in human heart and kidney transplantation. (A) Schematic overview of the study design and analytical workflow. (B) MEK–ERK pathway scores in human heart transplant biopsies (GSE150059) and kidney transplant biopsies (GSE21374), comparing stable and rejection samples. (C) Kaplan–Meier curves showing renal allograft survival in high‐ and low‐MEK–ERK pathway groups (top/bottom 30%). Statistical significance for panel (B) was determined using the Mann–Whitney *U* test, the log‐rank test for (C). ns, not significant (*p* > .05); **p < .01; ***p < .001; ****p < .0001.

## 4 DISCUSSION

Elucidating the mechanisms underlying T cell activation and effector function remains a central challenge in transplant immunology.^27^, ^33^, ^34^, ^35^ In the present study, MEK1/2 signalling emerged as a key modulator of T cell–mediated acute cardiac allograft rejection. Single‐cell transcriptomic analysis revealed pronounced activation of the MEK/ERK pathway in effector CD4^+^ and CD8^+^ T cells infiltrating recipient spleens and grafts during acute rejection, which was further confirmed via flow cytometry and immunofluorescence. Functionally, T cell‐specific *Mek1/2* deletion and pharmacological MEK1/2 inhibition suppressed T cell activation and effector function, leading to attenuated rejection and prolonged graft survival. Consistent with these findings, inhibition of ERK signalling reduces T cell activation, and MEK inhibitors selectively suppress alloreactive T cells. Figure 8 shows a schematic of the proposed mechanism.

**FIGURE 8:**
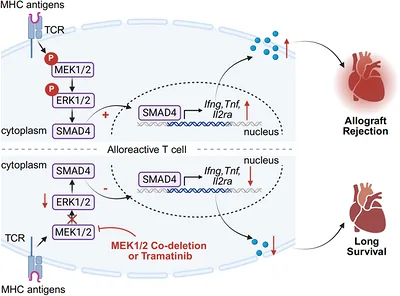
Upon T cell receptor (TCR) engagement by alloantigens, MEK1/2–ERK1/2 signalling promotes SMAD4 nuclear translocation and supports SMAD4‐dependent transcription of effector genes, including *Ifng, Tnf*, and *Il2ra*. This transcriptional program promotes alloreactive T cell effector function and contributes to cardiac allograft rejection. Conversely, T cell‐specific *Mek1/2* double‐knockout or pharmacological MEK1/2 inhibition with trametinib reduces SMAD4 nuclear translocation, attenuates effector gene expression, and ultimately prolongs cardiac allograft survival.

Mechanistically, TCR engagement activates multiple interconnected signalling pathways, including the RASGRP1–RAS–MEK‐ERK1/2–AP1, p38/JNK, and TSC1/2–mTOR pathways. Among these, the MEK–ERK axis serves as a key link between TCR signalling and transcriptional regulation, with ERK1/2 activity closely coupled to TCR signal strength and contributing to c‐Fos/AP‐1 activation, IL‐2 production, proliferation, and effector differentiation.^15^, ^36^, ^37^, ^38^, ^39^, ^40^ However, its functional requirement is context‐dependent and varies across T cell subsets and activation states.^41^, ^42^ For example, ERK activation is required for the upregulation of Foxp3 and the acquisition of suppressive activity in regulatory T cells.^43^ Other signalling branches can provide additional or compensatory inputs: TCR‐induced p38 activation can occur through a noncanonical pathway downstream of proximal TCR signalling, whereas JNK activation is mediated, at least in part, through the PKCθ and CBM complex.^44^, ^45^ In parallel, both RAS–ERK1/2 and PI3K–AKT signalling contribute to mTORC1/2 activation and influence CD4^+^ T cell differentiation.^46^, ^47^ Consistent with this pathway crosstalk, MEK inhibition has been shown to enhance AKT and p38/JNK/NF‐κB signalling, with increased p38/JNK activity partially restoring T cell cytokine production.^38^, ^48^ In contrast, Tpl2–MEK–ERK signalling disruption in memory and effector CD8^+^ T cells can markedly reduce MEK–ERK activation without coordinated changes in other signalling pathways,^49^ highlighting the distinct but interconnected regulation of these signalling branches.

In this broader network, our findings identify MEK1/2 as an important signalling node in the effector response of alloreactive T cells. Using a T cell‐specific *Mek1/2* double‐knockout model (*Map2k1^fl/fl^ Map2k2^fl/fl^ CD4‐Cre^+^*), it was demonstrated that the loss of MEK1/2 signalling markedly impaired T cell expansion, activation, and effector molecule production in vitro while attenuating acute rejection in vivo. These findings further expand the current understanding of MEK1/2 as a key signalling node that integrates activation and metabolic cues to sustain effector T cell responses. Previous studies have shown that MAPK signalling also contributes to thymic T cell development, suggesting that sustained disruption of MEK1/2 signalling may exert developmental effects.^50^ Consistent with this possibility, we observed a modest reduction in the CD4^+^ T cell compartment in both the thymus and spleen in our T cell‐specific *Mek1/2* knockout model.^29^ However, these developmental changes were relatively limited and are unlikely to fully explain the pronounced defects in T cell activation and effector function observed following stimulation.

Phosphoproteomic analysis further revealed that *Mek1/2* deficiency substantially altered the SMAD2/3:SMAD4 signalling axis. In the present study, we showed that MEK1/2 deficiency impairs SMAD4 nuclear translocation in activated T cells, as demonstrated by immunofluorescence and western blotting analyses, suggesting a mechanistic association between upstream kinase signalling and downstream transcriptional regulation. However, the incomplete inhibition of SMAD4 nuclear localization following MEK1/2 deficiency indicates that MEK1/2 is likely an important but nonexclusive regulator of SMAD4 activation. Indeed, SMAD4 nuclear translocation is regulated by both TGF‐β‐dependent and TGF‐β‐independent mechanisms,^24^, ^51^, ^52^, ^53^ and multiple signalling pathways downstream of TCR activation, including MEK/ERK and PI3K signalling, have been implicated in controlling SMAD4 activity. Consistent with previous evidence linking MEK1/2‐mediated regulation of SMAD4 nuclear localization to CD8^+^ T cell cytotoxicity, our retroviral SMAD4 overexpression experiments further support a potential role of the MEK1/2–SMAD4 axis in shaping effector T cell responses. Moreover, SMAD4 function in T cells is highly context‐ and subset‐dependent and can extend beyond canonical TGF‐β signalling.^53^ In Th17 differentiation, SMAD4 can coordinate with SKI to suppress RORγt expression and restrain Th17 development, whereas TGF‐β promotes Th17 differentiation by relieving this SMAD4‐mediated repression.^54^ In contrast, SMAD4 can also exert TGF‐β‐independent functions in naïve CD8^+^ T cells, where it epigenetically preconditions T cell fate and regulates their subsequent responsiveness to TGF‐β.^30^ These findings indicate that the functional output of SMAD4 signalling may depend on the T cell subset, activation state, and the upstream signalling environment. In support of a potential role for SMAD4 in effector‐associated transcriptional programs, analysis of publicly available SMAD4 ChIP‐seq datasets revealed enrichment at genomic regions related to T cell activation and effector function, including *Il2ra, Ifng, and Tnf*. These data provide supportive evidence that SMAD4 may participate in transcriptional programs that regulate T cell activation and cytokine production.^55^ Overall, our findings suggest that MEK1/2‐associated regulation of SMAD4 may contribute to the effector program of alloreactive T cells, further highlighting the context‐dependent nature of SMAD4 function in T cell responses.

As trametinib, a selective MEK1/2 inhibitor, has already been approved by the FDA for cancer treatment,^56^ its therapeutic potential in acute allograft rejection was thoroughly evaluated. Pharmacological MEK1/2 inhibition significantly prolonged graft survival, reduced inflammatory infiltration, and diminished T cell cytokine production, consistent with the phenotypes observed in mice with T cell‐specific *Mek1/2* deletion. Supporting these findings, Chauveau et al.^57^ demonstrated that MEK inhibition protects against human skin graft rejection by enhancing CD8^+^TCF1^+^ stem‐like T cell formation while limiting terminal differentiation of CD8^+^ effector T cells, thereby shifting alloimmune responses towards a less‐pathogenic state. In parallel, ERK inhibition has also been reported to promote allograft engraftment via metabolic T cell reprogramming, highlighting the central role of the MEK/ERK signalling axis in coordinating immune activation with cellular metabolism.^15^ However, because trametinib systemically inhibits MEK1/2, its therapeutic effects may extend beyond T cells. MEK inhibition can modulate dendritic‐cell and regulatory B‐cell functions,^58^, ^59^ and MEK1/2 signalling also contributes to the immunometabolic programming of allograft‐infiltrating macrophages during cardiac transplant rejection.^25^ Thus, the therapeutic effects of trametinib may reflect both T cell‐intrinsic and broader immunomodulatory actions, while the concordant effects of pharmacological inhibition and T cell‐specific *Mek1/2* deletion support T cell MEK1/2 signalling as a key component of its therapeutic activity.

Furthermore, transcriptomic analyses of publicly available cardiac and renal allograft biopsy datasets revealed that increased MEK1/2 pathway activity was significantly correlated with acute rejection and inferior allograft survival, further supporting the clinical relevance of our findings. Although differences in inflammatory cell composition may influence bulk biopsy transcriptomes, the consistent association observed across independent cardiac and renal transplantation cohorts, along with our mechanistic findings in the murine model, strengthens the translational relevance of MEK1/2 signalling in allograft rejection. Therefore, these human transcriptomic analyses provide complementary clinical evidence without, by themselves, establishing a causal or cell type‐specific role for MEK1/2 signalling.

This study has several limitations that warrant consideration. First, our analysis mainly focused on bulk CD4^+^ and CD8^+^ T cell populations and did not specifically distinguish antigen‐specific T cells. Second, the findings were largely derived from mouse models, and the limited availability of clinical samples precluded systematic validation in human tissues. Therefore, future studies incorporating patient‐derived samples and longitudinal follow‐up cohorts will be important to establish clinical relevance. Third, genetic *Mek1/2* deficiency may affect thymic T cell development, as evidenced by the modest reduction in the CD4^+^ T cell compartment in the thymus and spleen. Although these changes are unlikely to fully explain the observed functional defects, inducible and temporally controlled *Mek1/2* deletion models will be valuable for further delineating the cell‐intrinsic role of MEK1/2 in mature T cells. Fourth, owing to technical constraints, direct assessment of SMAD4 phosphorylation sites was not performed, and our conclusions are primarily based on its nuclear translocation. Finally, our transplantation models were not specifically designed to distinguish direct from indirect presentation or to define the temporal contribution of MEK1/2 signalling to these distinct pathways. Further studies using experimental systems that selectively distinguish direct from indirect presentation are needed to determine whether MEK1/2 signalling has pathway‐ or stage‐specific functions during allograft rejection.

Overall, this study establishes the MEK1/2–SMAD4 axis as a crucial pathway that regulates T cell activation and effector function in cardiac allograft rejection. These findings elucidate how intracellular signalling networks coordinate alloimmune responses and support therapeutic targeting of this pathway to limit acute rejection and improve transplant outcomes.

## AUTHOR CONTRIBUTIONS

Y.L., J.C., and J.X. provided direction and guidance throughout the preparation of this manuscript; J.Z. and S.R. wrote and edited the manuscript. W.Z., J.C., and J.W. reviewed and made significant revisions to the manuscript. L.L. and S.Z. collected and prepared the related papers. S.Z. and J.Z. helped to draw the figures. All authors have read and approved the final manuscript.

## CONFLICT OF INTEREST STATEMENT

The authors declare no conflict of interest.

## ETHICS STATEMENT

All animal experiments in this study were reviewed and approved by the Institutional Animal Care and Ethics Committee of Huazhong University of Science and Technology (IACUC number 5041). All animal experiments were carried out in accordance with the guidelines of the NIH Guide for the Care and Use of Laboratory Animals

## Supporting information



Supporting Information

## Data Availability

The data that support the study findings are available upon reasonable request from the corresponding authors.
