# Supplementary material for: MEK1/2 Signalling establishes an alloreactive effector T cell program through SMAD4 during cardiac allograft rejection
Source: Clin Transl Med. 2026 Oct 27;16(11):e70865. doi: 10.1002/ctm2.70865 (PMC13714352; doi:10.1002/ctm2.70865)
Supplement: Supplementary file 1 [file CTM2-16-e70865-s001.docx]

**Supplemental Materials**

**MEK1/2 Signaling Establishes an Alloreactive Effector T Cell Program Through SMAD4 During Cardiac Allograft Rejection**

**This file includes:**

Supplementary Methods

Supplementary Table 1-3

Supplementary Figure 1-2

**Supplementary Materials and Methods**

**Methods**

**Histological and immunofluorescence analysis**

At the indicated time points after cardiac transplantation, grafts were harvested and fixed in 4% paraformaldehyde for 12–24 h. Samples were then dehydrated through graded ethanol, cleared in xylene, and embedded in paraffin. Paraffin blocks were sectioned into 4–5 μm slices.

The graft sections were deparaffinized in xylene, rehydrated through graded ethanol, and stained with hematoxylin and eosin (H&E). Histological images were acquired using an Olympus VS120 slide scanner (Olympus, Japan) and analyzed with CaseViewer (v2.4). Acute cellular rejection was evaluated according to the 2004 International Society for Heart and Lung Transplantation (ISHLT) guidelines^1^.

For immunofluorescence staining of cardiac grafts, the TSAPlus fluorescence staining kit (G1259, Servicebio) was used according to the manufacturer’s instructions. Briefly, mouse cardiac graft sections were deparaffinized and rehydrated, followed by antigen retrieval using an appropriate buffer. After cooling to room temperature, sections were washed three times with PBS. Sections were then permeabilized with 0.3% Triton X-100 and blocked with 3% bovine serum albumin (BSA). Primary antibodies were incubated overnight at 4°C. The following primary antibody was used: anti-CD3 (1:2000, GB13014, Servicebio); anti- phospho-p44/42 MAPK (Erk1/2) (Thr202/Tyr204) (1:200, 4370S, Cell Signaling Technology); anti-phospho-MEK1/2 (Ser218, Ser222, Ser226) (1:200, Invitrogen, 44-454G). Secondary antibodies conjugated to donkey anti-rabbit Alexa Fluor 488 (1:500, A-21206, Invitrogen) and donkey anti-mouse Alexa Fluor 594 (1:500, A-21207, Invitrogen) were used.

For immunofluorescence staining of T cells, confocal dishes were pre-coated with 0.1 mg/mL poly-L-lysine (C0313, Beyotime), and T cells were seeded and allowed to adhere at 37°C for 1–2 h. Cells were then fixed with 4% paraformaldehyde for 15 min, permeabilized with 0.3% Triton X-100 for 15 min, and blocked with 5% BSA for 1 hours. Cells were incubated with anti-SMAD4 antibody (1:250; 17387-1-AP, Proteintech) overnight at 4°C, followed by incubation with Cy3-conjugated goat anti-rabbit secondary antibody (1:500; A0516, Beyotime) for 1 h at room temperature. Nuclei were counterstained with DAPI (2 μg/mL; AC28L533, Beyotime) for 10 min in the dark. The proportion of cells exhibiting higher nuclear than cytoplasmic SMAD4 fluorescence intensity was manually quantified to assess nuclear translocation.

Fluorescence images were acquired using an Olympus FV3000 confocal laser scanning microscope (Olympus, Japan) and analyzed with CaseViewer (v2.4).

**Single-cell suspension preparation**

Single-cell suspensions from spleens were prepared by mechanical dissociation and passed through a 40 μm cell strainer. Red blood cells (RBCs) were lysed using ACK lysis buffer (A1049201, Gibco). Cells were then centrifuged (2,000 rpm, 3 min) and washed twice with cold PBS.

Cardiac tissues were minced into small fragments and digested in Hanks’ balanced salt solution containing calcium and magnesium supplemented with collagenase B (1 mg/mL; 11088807001, Merck) at 37°C for 30 min. The digested tissue was filtered through a 70 μm cell strainer to obtain single-cell suspensions. Cells were then resuspended in 40% Percoll and subjected to density gradient centrifugation at 600 × *g* to enrich lymphocytes. Red blood cells were lysed using ACK lysis buffer, followed by washing with PBS. Cells were collected for subsequent analyses.

**Immunoblot analysis**

Activated cells were lysed using a Nuclear and Cytoplasmic Protein Extraction Kit (P0027, Beyotime) supplemented with protease and phosphatase inhibitor cocktails (GRF103, Epizyme) according to the manufacturer’s instructions. Proteins were separated by SDS–PAGE and transferred onto 0.22 μm PVDF membranes (Millipore, Billerica, MA, USA). Membranes were blocked with 5% BSA and incubated with primary antibodies against SMAD4 (1:1000; 17387-1-AP, Proteintech) overnight at 4°C. After washing, membranes were incubated with horseradish peroxidase (HRP)-conjugated goat anti-rabbit secondary antibodies. Band intensities were quantified using ImageJ software, and the relative expression of SMAD4 was normalized to GAPDH (cytoplasmic fraction) or Histone H3 (nuclear fraction) to evaluate its nuclear–cytoplasmic distribution.

**Retroviral transduction**

For retroviral transduction, naïve T cells were enriched from the spleens using a Pan T Cell Isolation Kit II (130-095-130; Miltenyi Biotec). Briefly, cells were resuspended at 1 × 10⁶ cells/mL in complete RPMI and activated on plate-bound anti-CD3 and anti-CD28 for 24 hours. Activated T cells were then resuspended in a mixture of fresh complete RPMI and retroviral supernatant supplemented with soluble anti-CD3, anti-CD28, IL-2 (50 U/mL), and Polybrene (5ug/ml; Sigma-Aldrich). The cell suspension was transferred to 6-well plates and centrifuged at 800 × g for 90 minutes at 30℃. The retroviral transduction procedure was repeated the following day. Transduced T cells were cultured for 2 days, replacing RPMI and adding fresh IL-2 (50 U/mL). Retroviral supernatants were generated by transfecting HEK-293T cells with the indicated retroviral expression plasmid, including SMAD4 cloned into the pMP71 retroviral vector, together with the packaging plasmid pCL-Eco.

**4D-DIA phosphoproteomics analysis**

4D-DIA phosphoproteomics analysis was performed by oebiotech (Shanghai, China).

Mouse naïve T cells were isolated from spleens of WT or *Map2k1^fl/f^Map2k2^fl/fll^Cd4-Cre^+^* mice by magnetic separation as described above. Cells were stimulated with anti-mouse CD3 (100302; Biolegend) and anti-CD28 (102102; Biolegend) for 3 days and activation efficiency (>90%) was confirmed by flow cytometry. Protein was extracted using lysis buffer supplemented with protease and phosphatase inhibitors (P1049, Beyotime), and concentrations were determined by Enhanced BCA Protein Assay Kit (P0009, Beyotime). Equal amounts of protein were reduced, alkylated, and digested with trypsin. The peptides were desalted using C18 solid-phase extraction column, followed by TiO₂-based enrichment of phosphopeptides and lyophilization. The enriched peptides were resuspended in 0.1% formic acid aqueous solution and subjected to nano-LC-MS/MS analysis using a four-dimensional data-independent acquisition (4D-DIA) strategy. Raw DIA data were processed using Spectronaut Pulsar 19.3 (Biognosys). Peptide and protein identification was performed with an FDR <0.01. Differentially phosphorylated sites were defined by a fold change ≥2 or ≤0.5 and *adjusted p* < 0.05.

**Single-cell RNA sequencing analysis**

The single-cell RNA sequencing (scRNA-seq) data for murine cardiac allograft rejection (GSE249989) were published by our laboratory^2^, was obtained from the GEO database ([https://www.ncbi.nlm.nih.gov/geo/](https://www.ncbi.nlm.nih.gov/geo/query/acc.cgi?acc=GSE249989)). Two additional published murine cardiac allograft rejection datasets (GSE151048 and PMID: 34870762) were included for independent validation. The dataset associated with PMID: 34870762 was obtained from Figshare (<https://figshare.com/s/11d8b3c99165c5fd8cd3>). The preprocessed gene expression matrix was analyzed in R (v4.3.0) using Seurat (v4.3.0). Cells with＜500 or＞5,000 detected genes, or with mitochondrial gene content >10%, were excluded. Data were normalized using *NormalizeData*, and 2,000 highly variable genes were identified using *FindVariableFeatures* with the “vst” method. After scaling with *ScaleData*, principal component analysis was performed using *RunPCA*. Batch effects were corrected using Harmony (v1.0.3), and the top 20 principal components were used for downstream analysis. Cell clustering was performed using *FindNeighbors* and *FindClusters,* and visualized using UMAP. Differentially expressed genes were identified using *FindAllMarkers*. Cell type annotation was performed using SingleR (v1.4.1) with the ImmGen database and visualized with *DimPlot*. Gene expression density was visualized using the Nebulosa package (v1.0.1). Gene set activity scores were calculated using *AddModuleScore* and visualized with the ggpubr (v0.6.0). Volcano plots and heatmaps were generated using scRNAtoolVis (v0.0.7) and *DoHeatmap*, respectively. For pathway analysis of effector T cells, differentially expressed genes were identified using a threshold of fold change >1.5 and adjusted *P* < 0.05, followed by KEGG and GSEA enrichment analyses.

**ChIP-seq Data Analysis**

The ChIP-seq dataset (GSE192620) was used to validate the regulatory role of SMAD4^3^ and was obtained from the GEO database (<https://www.ncbi.nlm.nih.gov/geo/query/acc.cgi?acc=GSE192620>). Clean reads after filtering were aligned to the mouse reference genome (mm10) using Bowtie2 (v2.5.1). Peak calling was performed using MACS2 (v2.2.7.1), and differential binding analysis was conducted with DiffBind (v3.10.1). Genome browser tracks were visualized using the Integrative Genomics Viewer (IGV).

**Statistical analysis**

All statistical analyses were performed using GraphPad Prism (version 9.2.0). Unless otherwise indicated, data are presented as mean ± standard deviation (SD). For comparisons between two groups, a two-tailed unpaired Student’s *t*-test was used for normally distributed data with equal variances, while Welch’s *t*-test was applied when variances were unequal. For non-normally distributed data, the Mann–Whitney *U* test was used. For comparisons among multiple groups, one-way or two-way ANOVA was used for normally distributed data, whereas the Kruskal–Wallis test was applied for non-normally distributed data, followed by Dunnett’s or Sidak’s post hoc multiple comparisons tests, as appropriate. Statistical significance is indicated in the figure legends as follows: ns, not significant; **p* < 0.05; ***p* < 0.01; ****p* < 0.001; *****p* < 0.0001.

**Supplemental Tables**

**Table S1: Flow cytometry antibodies**

| **Antibodies** | **Source** | **Identifier** |
| --- | --- | --- |
| FITC anti-mouse CD62L Antibody | BioLegend | 161212 |
| PerCP/Cyanine5.5 anti-mouse/human CD44 Antibody | BioLegend | 103032 |
| APC anti-mouse CD45 Antibody | BioLegend | 103112 |
| FITC anti-mouse CD8a Antibody | BioLegend | 100706 |
| Brilliant Violet 421™ anti-mouse CD4 | BioLegend | 100443 |
| PE-cy7 anti-mouse Foxp3 antibody (FJK-16s) | eBioscience | 25-5773-82 |
| Brilliant Violet 605™ anti-mouse CD45 | BioLegend | 103155 |
| APC anti-mouse TCR-β | BioLegend | 109212 |
| PE anti-mouse/human CD44 antibody (IM7) | BioLegend | 103008 |
| APC/Cyanine7 anti-mouse CD62L Antibody | BioLegend | 104428 |
| APC/Cyanine7 anti-mouse CD8a Antibody | BioLegend | 100714 |
| PE-cy7 anti-mouse IFN-γ antibody | BioLegend | 505825 |
| APC anti-mouse TNF-α Antibody | BioLegend | 506308 |
| FITC anti-mouse Perforin Antibody | BioLegend | 154310 |
| Brilliant Violet 421™ anti-human/mouse Granzyme B Recombinant Antibody | BioLegend | 396414 |
| AF700 anti-mouse CD8a antibody | BioLegend | 100730 |
| APC anti-mouse CD25 Antibody | BioLegend | 113709 |
| Brilliant Violet 421™ anti-mouse CD279 (PD-1) | BioLegend | 135218 |
| PE-cy7 anti-mouse Ki-67 antibody | BioLegend | 652425 |
| PE anti-human/mouse Granzyme B Recombinant Antibody | BioLegend | 372208 |
| PE Annexin V Apoptosis Detection Kit with 7-AAD | BioLegend | 640934 |
| PE anti-mouse CD4 antibody | BioLegend | 100408 |
| PE-cy7 anti-mouse CD8a antibody | BioLegend | 100722 |
| FITC anti-mouse TCR-βantibody | BioLegend | 109206 |
| APC-cy7 anti-mouse CD4 antibody | BD Biosciences | 552051 |
| Phospho-p44/42 MAPK (Erk1/2) (Thr202/Tyr204) (E10) Mouse Monoclonal Antibody (Alexa Fluor® 647 Conjugate) | Cell Signaling Technology | 43755 |
| Phospho-MEK1/2 (Ser221) (166F8) Rabbit Monoclonal Antibody (PE Conjugate) | Cell Signaling Technology | 16211S |
| Zombie Aqua™ Fixable Viability Kit | BioLegend | 423102 |

**Table S2: Oligonucleotide** **sequences**

| **Mouse genotype primer sequences** | |
| --- | --- |
| *Map2k1^fl/fl^* | Forward: TGGCCTCGAACTCAGAAATCCA |
|  | Reverse: TAGAAGGAACGGCACAATACAAAC |
| *Map2k2^fl/fl^* | Forward: CCGGTGCTGCTGAGGAAGGA |
|  | Reverse: CCAAGAACCAGGCCCAAGAGG |

**Table S3: Western blot antibodies and Immunofluorescent antibodies.**

| **Antibodies** | **Source** | **Identifier** |
| --- | --- | --- |
| SAMD4A Polyclonal antibody | Proteintech | 17387-1-AP |
| GAPDH Polyclonal antibody | Proteintech | 10491-1-AP |
| HRP-conjugated Affinipure Goat Anti-Rabbit IgG | Proteintech | SA00001-2 |
| Phospho-p44/42 MAPK (Erk1/2) (Thr202/Tyr204) | Cell Signaling Technology | 4370S |
| Phospho-MEK1/2 (Ser221) | Affinity Biosciences | AF3385 |
| Anti-CD3 Rabbit mAb | Servicebio | GB13014 |
| Donkey anti-Rabbit IgG (H+L) Highly Cross-Adsorbed Secondary Antibody, Alexa Fluor™ 488 | Invitrogen | A-21206 |
| Donkey anti-Rabbit IgG (H+L) Highly Cross-Adsorbed Secondary Antibody, Alexa Fluor™ 594 | Invitrogen | A-21207 |
| Cy3-labeled Goat Anti-Rabbit IgG (H+L) | Beyotime | A0516 |

**Supplemental Figures and Figure Legends**

**Supplemental Figure 1**

**Supplemental Figure 1 scRNA-seq library construction and analysis.**
(A) Schematic of the murine heart transplantation model and immune cell isolation procedures. (B) Workflow of scRNA-seq data analysis, including quality control and standardization, dimensionality reduction and clustering, comparative analysis of T-cell subsets, differential gene expression analysis, KEGG and GSEA pathway enrichment analyses, and gene signature scoring. (C) Bar chart showing the proportions of the three groups in different T cell subsets. (D) Scatter plot illustrating DEGs between Allo_graft Teff CD8 and Allo_spleen Naïve CD8 T cell subsets. Upregulated (red dots) and downregulated (blue dots) DEGs are highlighted. (E) Dot plot showing significantly enriched KEGG pathways among DEGs between Allo_graft Teff CD8 and Allo_spleen Naïve CD8 T cell subset clusters. (F) Gene set enrichment analysis plots of MEK/ERK pathway–related genes in Teff CD8 cells.

**Supplemental Figure 2**

**Supplemental Figure 2 Flow cytometry gating strategies for recipient mice on postoperative day 6 after heart transplantation.**
(A) Gating strategy for splenic T-cell subsets. (B) Gating strategy for CD44^+^CD62L^−^ cells within splenic CD4^+^ and CD8^+^ T cells. (C) Gating strategy for TNF-α^+^IFN-γ^+^ cells within splenic CD4^+^ and CD8^+^ T cells. (D) Gating strategy for GZMB^+^Perforin^+^ cells within splenic CD4^+^ and CD8^+^ T cells.

**References**

1.	Stewart S, Winters GL, Fishbein MC, Tazelaar HD, Kobashigawa J, Abrams J, et al. Revision of the 1990 working formulation for the standardization of nomenclature in the diagnosis of heart rejection. J Heart Lung Transplant. 2005;24(11):1710-20.

2.	Chen Z, Li Y, Niu Y, Zhang X, Yu J, Cui J, et al. MEK1/2-PKM2 Pathway Modulates the Immunometabolic Reprogramming of Proinflammatory Allograft-Infiltrating Macrophages During Heart Transplant Rejection. Transplantation. 2024.

3.	Liu X, Hao J, Wei P, Zhao X, Lan Q, Ni L, et al. SMAD4, activated by the TCR-triggered MEK/ERK signaling pathway, critically regulates CD8(+) T cell cytotoxic function. Sci Adv. 2022;8(30):eabo4577.
